# Risk Factors for Granulocytopenia in Patients with Graves' Disease Receiving Antithyroid Drugs

**DOI:** 10.1155/2023/9935195

**Published:** 2023-04-10

**Authors:** Jiaxi Li, Xiaowen Zhang, Lintong Li, Qiaoling Zhu, Weihong Ge, Cheng Ji

**Affiliations:** ^1^Department of Pharmacy, China Pharmaceutical University Nanjing Drum Tower Hospital, Nanjing, China; ^2^Nanjing Medical Center for Clinical Pharmacy, Nanjing, China; ^3^Department of Endocrinology, Nanjing Drum Tower Hospital, The Affiliated Hospital of Nanjing University Medical School, Nanjing, China; ^4^Department of Pharmacy, Nanjing Drum Tower Hospital, The Affiliated Hospital of Nanjing University Medical School, Nanjing, China

## Abstract

**Objective:**

To study the risk factors for granulocytopenia caused by antithyroid drugs.

**Methods:**

Patients who were diagnosed with Graves' hyperthyroidism and regularly treated with antithyroid drugs (ATDs) from January 2010 to July 2022 at Nanjing Drum Tower Hospital, aged >18 years, were selected for general information and laboratory tests and divided into two groups according to the occurrence of granulocytopenia. Independent risk factors for the development of granulocytopenia in patients treated with ATDs were analyzed using one-way and multiway logistic regression analyses, and the predictive value of each index was evaluated using the receiver operating characteristic (ROC) curve and the area under the curve (AUC).

**Results:**

A total of 818 patients were enrolled, of which 95 developed granulocytopenia. Univariate analysis revealed that sex, white blood cell (WBC) counts, neutrophil-to-lymphocyte ratio (NLR), glutamic-pyruvic transaminase (ALT), aspartate transaminase (AST), free triiodothyronine (FT3), free thyroxine (FT4), and thyroid stimulating hormone (TSH) before medication were risk factors for ATD-induced granulocytopenia (*P* < 0.05). The abovementioned indicators were taken as independent variables, and multivariate logistic regression analysis showed that female sex, higher ALT levels before medication, and lower NLR and WBC levels were independent risk factors for granulocytopenia using ATDs (*P* < 0.05). ROC curve analysis showed that sex, NLR, ALT, and WBC count had significant predictive values (*P* < 0.05), and NLR and WBC count had higher predictive values (AUC = 0.916 and 0.700, respectively).

**Conclusion:**

Sex, NLR, ALT, and WBC were the main risk factors for granulocytopenia in patients with ATD.

## 1. Introduction

The prevalence of hyperthyroidism is 0.2–1.3% in regions with sufficient iodine supply [1], and recent data have shown that the prevalence of hyperthyroidism in China is up to 0.78% [2]. The causes of hyperthyroidism include Graves' disease (GD), toxic multinodular goiter, and toxic thyroid adenoma, among which GD is the most common. The major treatments for GD include antithyroid drugs (ATD), 131I therapy, and surgery. ATD is the primary treatment of GD. Long-term ATD therapy is cost-effective, has favorable efficacy without damaging thyroid tissues, and causes few complications. It presents a certain advantage in improving patients' quality of life and other biological outcomes [3]. However, adverse drug reactions (ADR) may occur during administration, including pruritus, liver injury, granulocytopenia, and agranulocytosis. Granulocytopenia is defined as a neutrophil count (NEUT) <2.0 × 10 ^ 9/L. Agranulocytosis is defined as NEUT <0.5 × 10 ^ 9/L, commonly manifests as sore throat, high fever, and pneumonia; skin infections or sepsis may occur in severe cases [4]. Agranulocytosis usually occurs 2–3 months after administration and has a low incidence (about 0.1–0.5%), but once it develops, the mortality rate can be up to 4.0–6.3% [5]. Therefore, patients who developed granulocytopenia during the treatment of GD with ATD were included to further study the risk factors. Raising awareness of this group of patients in clinical practice, further preventing granulocyte deficiency by predicting risk factors, and providing a reference for the early detection of high-risk patients, are the issue to be explored in this study.

## 2. Materials and Methods

### 2.1. Research Subjects

We retrospectively examined that patients who were diagnosed with GD and treated with ATD (Department of Endocrinology, Nanjing Drum Tower Hospital, January 2010–July 2022) were recruited as subjects.

The inclusion criteria were as follows: (1) patients diagnosed with Grave's disease according to the *Chinese Guidelines for diagnosis and Management of Hyperthyroidism and Other Causes of*Thyrotoxicosis [5]; (2) patients receiving regular antithyroid therapy with methimazole (MMI) and propylthiouracil (PTU) for more than a week; (3) no abnormality observed in patients' NEUT prior to ATD therapy.

The exclusion criteria were as follows: (1) patients with a previous history of immune system and hematologic diseases; (2) granulocytopenia due to exposure to radiation toxins, chemoradiotherapy, or antibody therapy; (3) acute granulocytopenia (also known as transient granulocytopenia) caused by infection, inflammation, etc.; (4) patients who did not receive drugs regularly or switched to surgery or 131I therapy halfway through the treatment; (5) patients with large amounts of missing data.

### 2.2. Research Methods

General information and laboratory examinations: General information and laboratory indicators of the subjects were retrospectively collected using the medical record system of Nanjing Drum Tower Hospital. General information included sex, age, ATD type, initial drug dose (low dose: methimazole ≤20 mg/d, propylthiouracil ≤200 mg/d; high dose: methimazole ≥20–30 mg/d, propylthiouracil ≥200–300 mg/d), and duration of ATD treatment. Laboratory indicators included NEUT, white blood cell count (WBC), neutrophil-to-lymphocyte ratio (NLR), monocyte-to-lymphocyte ratio (MLR), platelet-to-lymphocyte ratio (PLR), mean platelet volume (MPV), alanine aminotransferase (ALT), aspartate aminotransferase (AST), free triiodothyronine (FT3), free thyroxine (FT4), serum triiodothyronine (TT3), serum total thyroxine (TT4), thyroid stimulating hormone (TSH), antithyroid peroxidase antibody (TPOAb), antithyroglobulin antibody (TgAb), and antithyrotropin receptor antibody (TRAb).

Grouping: Patients were assigned to observation and control groups based on the occurrence of granulocytopenia. Granulocytopenia (observation group) was defined as NEUT <2.0 *∗* 10 ^ 9/L in routine blood tests during ATD treatment, and patients with granulocytopenia induced by malignancy, immune system diseases, hematologic diseases, or other drugs were excluded. Patients in the control group did not present with granulocytopenia during ATD administration.

### 2.3. Statistical Analysis

Statistical analysis was performed using SPSS.26.0. Normally, distributed continuous data were expressed as mean values ± standard deviations (SD), and comparisons between groups were conducted using parametric tests. Nonnormally distributed continuous data were expressed as median values and interquartile range (IQR), and comparisons between groups were performed using nonparametric tests. Categorical data were expressed as *n* (%) and analyzed using the *χ*^2^ test. Significant indicators in the univariate analysis were included in the multivariate logistic regression analysis to determine the factors for granulocytopenia in patients receiving ATD, with the adjusted OR values and 95% CI calculated. Receiver operating characteristic (ROC) curves were plotted, and the area under the curve (AUC) was acquired. AUC >0.5 represented predictive value, and *P* < 0.05 stood for statistical significance.

## 3. Results

### 3.1. General Information Analysis

Altogether, 818 patients (306 males and 512 females) were analyzed. The average age was 52.5 ± 17.1 years old (range 18–94 years). A total of 706 patients received MMI treatment and 112 patients received PTU treatment. Among them, 95 patients (25 men and 70 women) showed granulocytopenia (11.6%). The duration of ATD treatment was 8–96 days. Complete blood cell tests ≥2 times per patient. The patient's red blood cell count was (4.44 ± 0.7) × 10^12/L, WBC count 5.8 (4.7–7.3) × 10 ^ 9/L, and platelet count was 201 (165–250) × 10 ^ 9/L.

### 3.2. Univariate Analysis of Factors for Granulocytopenia Induced by ATD

Univariate analysis showed that no significant differences existed in age, drug type, initial drug dose, MLR, PLR, MPV, TT3, TT4, TPOAb, TgAb, and TRAb between the two groups (*P* > 0.05), whereas differences in sex, WBC, NLR, ALT, AST, FT3, FT4, and TSH levels between the two groups were significant (*P* < 0.05) (Table 1).

### 3.3. Multivariate Analysis of Factors for Granulocytopenia Induced by ATD

The occurrence of granulocytopenia was regarded as the dependent variable, and the eight significant factors in the univariate analysis were included as independent variables. Binary logistic regression analysis was performed after assigning values to variables. Male was assigned a value of 0 and female was assigned a value of 1. According to the low value below the normal range, the normal range, and the high value above the normal range, the values of WBC, NLR, FT3, FT4, and TSH were divided into three categories. ALT and AST were divided into two categories with normal values as the dividing line, with values of 0 and 1, respectively. The results showed that sex, NLR, ALT, and WBC count were independent risk factors for granulocytopenia; female sex and ALT elevation were risk factors; NLR and WBC elevation were protective factors (Table 2).

### 3.4. The Diagnostic Values of Gender, ALT, WBC, and NLR

Females and higher ALT indicated positive results, that is, granulocytopenia, presenting low diagnostic values; lower NLR and WBC indicated positive results. NLR had an excellent predictive performance for granulocytopenia in patients receiving ATD (AUC = 0.916, sensitivity = 89.5, specificity = 80.4). The results of the ROC analysis are shown in Table 3.

## 4. Discussion

Granulocytopenia occurs in approximately 10% of newly diagnosed and untreated GD patients [5]. ATD does not need to be discontinued when NEUT >1.5 *∗* 10 ^ 9/L and granulocyte count could recover after administering WBC-elevating drugs for a certain period. However, inappropriate management can lead to further development of agranulocytosis, which greatly increases the risk of infection and can even be life-threatening. Therefore, it is crucial to analyze risk factors for ATD-induced granulocytopenia.

### 4.1. Risk Factors for Granulocytopenia in Patients Using ATD

#### 4.1.1. Gender

This study revealed that sex might be a risk factor for ATD-induced granulocytopenia. A retrospective study in Japan showed that among all patients with Graves' disease [6], a greater number of women were treated with ATD to develop granulocytopenia (*p* < 0.05). Although hyperthyroidism is more common in females, adverse reactions caused by drugs are more related to individual factors, such as physiological conditions and genetic factors. Research has shown that females have almost double the risk of ADR compared with males, possibly because different populations have different pharmacokinetics [7]. Therefore, the effect of gender on ATD-induced granulocytopenia requires further investigation.

#### 4.1.2. ALT

Reactive metabolites are produced during the ATD oxidation process to activate inflammasomes to induce immune responses, thereby destroying neutrophils [8]. This oxidation process is mediated by myeloperoxidase and cytochrome P450, which might explain why some drugs that induce granulocytopenia or agranulocytosis are hepatotoxic [4]. The current study demonstrated that patients with elevated ALT levels were more likely to have agranulocytosis; however, further investigations are required to validate this finding.

#### 4.1.3. WBC

Granulocytes are a type of WBC, and thyrotoxicosis can lead to reductions in the WBC count and granulocytes [9]. However, some studies have suggested that routine WBC monitoring may be the most effective predictor of agranulocytosis, secondary to ATD [10]. Studies from Japan also recommended WBC monitoring once every two weeks [11]. Multivariate regression analysis confirmed that reduction in WBC count was a risk factor for granulocytopenia. There is still no consensus about the necessity of regularly monitoring WBC and NEUT for early identification of ADR, but the guidelines recommend routine monitoring. Patients should be advised to discontinue ATDs if they develop fever, sore throat, or mouth ulcers during treatment and to have their blood tested immediately.

#### 4.1.4. NLR

The NLR is an inflammatory marker used as a prognostic indicator for the recurrence and survival of patients with cancer [12]. Meanwhile, research has shown that NLR can assist in the differentiation of hyperthyroidism with different etiologies [13]. In this study, the NLR was included as a variable. The ROC curves showed that NLR performed well in predicting granulocytopenia with higher sensitivity and specificity than sex, ALT, WBC count, etc. Therefore, close attention should be paid to NLR when administering ATD to avoid aggravation of granulocytopenia.

Taken together, sex, ALT, WBC, and NLR are significant predictors of ATD-induced granulocytopenia. Early identification of granulocytopenia is conducive to improving patient prognosis, reducing drug-induced ADR, and offering a reference for devising timely protocols in clinical practice. Due to the retrospective nature of the study, some data were missing, resulting in a small sample size available for analysis; the low incidence of ATD-induced granulocytopenia led to the low number of patients in the observation group available for analysis. Hence, a prospective study with a large sample size is required to further probe the risk factors for ATD-induced granulocytopenia, and mechanisms should be delved into to provide theoretical support for early diagnosis and prevention.

## Figures and Tables

**Table 1:** Univariate analysis of factors for granulocytopenia induced by antithyroid drugs.

Variables included	Observation group (*n*, %)	Control group (*n*, %)	*P* value
Gender (female)	70 (73.7)	442 (61.2)	0.017
WBC (×10^9^/L)	4.6 (3.90–6.00)	5.90 (5.00–7.40)	<0.001
NLR	0.89 (0.76–1.13)	2.05 (1.50–3.09)	<0.001
ALT ≥40 (IU/L)	34 (36.2)	128 (18.2)	<0.001
AST ≥40 (IU/L)	20 (21.1)	74 (10.5)	0.003
FT3 (pmol/L)	17.50 (9.02–30.13)	8.13 (4.66–15.78)	<0.001
FT4 (pmol/L)	0.01 (0.01-0.01)	0.01 (0.01–0.42)	<0.001
TSH (mIU/L)	0.01 (0.01-0.01)	0.01 (0.01–0.42)	<0.001

WBC: white blood cell counts, NLR: neutrophil-to-lymphocyte ratio, ALT: alanine aminotransferase, AST: aspartate aminotransferase, FT3: free triiodothyronine, FT4: free thyroxine, and TSH: thyroid stimulating hormone.

**Table 2:** Multivariate logistic regression analysis for granulocytopenia induced by antithyroid drugs.

Factors	*β*	S.E.	Wald	OR (95% CI)	*P*
*Gender*					
Male				1.000	
Female	0.826	0.314	6.918	2.285 (1.234, 4.229)	0.009

*ALT (IU/L)*					
<40				1.000	
≥40	1.158	0.321	13.023	3.183 (1.697, 5.968)	<0.001

*WBC (×10* ^*9*^ */L)*					
<4				1.000	
4–10	−2.068	0.369	31.459	0.126 (0.061, 0.26)	<0.001
>10	−2.266	0.842	7.244	0.104 (0.02, 0.54)	0.007

*NLR*					
<1				1.000	
1–3	−3.3	0.312	111.69	0.037 (0.02, 0.068)	<0.001
>3	−21.765	2874.561	0	0 (0.000)	0.994
Constant	1.515	0.471	10.363	4.549	0.001

ALT: alanine aminotransferase, WBC: white blood cell counts, and NLR: neutrophil-to-lymphocyte ratio.

**Table 3:** Receiver operating characteristic curve analysis.

Variables	AUC	*P* value	95% CI	Cut off	Sensitivity (%)	Specificity (%)
Gender	0.563	0.047	0.503–0.623			
ALT	0.630	<0.001	0.569–0.692	0.225	61.7	60.8
NLR	0.916	<0.001	0.888–0.944	0.699	89.5	80.4
WBC	0.700	<0.001	0.638–0.763	0.404	66.3	74.1

ALT: alanine aminotransferase, NLR: neutrophil-to-lymphocyte ratio, and WBC: white blood cell counts.

## Data Availability

The data used to support the findings of this study are available from the corresponding author upon request.
